# Endemic Burkitt lymphoma: a complication of asymptomatic malaria in sub-Saharan Africa based on published literature and primary data from Uganda, Tanzania, and Kenya

**DOI:** 10.1186/s12936-020-03312-7

**Published:** 2020-07-28

**Authors:** Lawrence S. Redmond, Martin D. Ogwang, Patrick Kerchan, Steven J. Reynolds, Constance N. Tenge, Pamela A. Were, Robert T. Kuremu, Nestory Masalu, Esther Kawira, Isaac Otim, Ismail D. Legason, Herry Dhudha, Leona W. Ayers, Kishor Bhatia, James J. Goedert, Sam M. Mbulaiteye

**Affiliations:** 1 Division of Cancer Epidemiology and Genetics, National Cancer Institute, National Institutes of Health, Bethesda, MD USA; 2 EMBLEM Study, St. Mary’s Hospital Lacor, Gulu, Uganda; 3 African Field Epidemiology Network, Kampala, Uganda; 4 EMBLEM Study, Kuluva Hospital Kuluva, Arua, Uganda; 5 Division of Intramural Research, National Institute of Allergy and Infectious Diseases, National Institutes of Health, Bethesda, MD USA; 6 EMBLEM Study, Moi University College of Health Sciences, Eldoret, Kenya; 7 EMBLEM Study, Academic Model Providing Access to Healthcare (AMPATH), Eldoret, Kenya; 8 EMBLEM Study, Bugando Medical Center, Mwanza, Tanzania; 9 EMBLEM Study, Shirati Health and Educational Foundation, Shirati, Tanzania; 10 Department of Pathology, The Ohio State University, Columbus, OH USA

**Keywords:** Burkitt lymphoma (BL), Malaria complications, *Plasmodium falciparum*, Epstein-barr virus (EBV), East Africa

## Abstract

**Background:**

Endemic Burkitt lymphoma (eBL) is an aggressive B cell non-Hodgkin lymphoma associated with antigenic stimulation from *Plasmodium falciparum* malaria. Whether eBL risk is related to malaria parasite density is unknown. To address this issue, children with eBL, asymptomatic and clinical malaria, as a surrogate of malaria parasite density, were assessed.

**Methods:**

Malaria-related laboratory results (parasite density, haemoglobin, platelet count, and white cell count [WBC]) count) were compiled for 4019 eBL cases and 80,532 subjects evaluated for asymptomatic malaria or clinical malaria (severe malaria anaemia, hyperparasitaemia, cerebral malaria, malaria prostration, moderate malaria, and mild malaria) in 21 representative studies published in Africa (mostly East Africa) and 850 eBL cases and 2878 controls with primary data from the Epidemiology of Burkitt Lymphoma in East African Children and Minors (EMBLEM) case–control study in Uganda, Tanzania, and Kenya. The average values of malaria-related laboratory results were computed by condition and trends across single-year age groups were assessed using regression and spline models.

**Results:**

Overall, malaria infection or malaria was diagnosed in 37,089 of children compiled from the literature. Children with eBL and asymptomatic parasitaemia/antigenaemia, but not those with clinical malaria, were closest in their mean age (age 7.1–7.2 vs. 7.4–9.8 years), haemoglobin level (10.0–10.4 vs. 11.7–12.3 g/dL), malaria parasite density (2800 vs. 1827–7780 parasites/µL), platelet count (347,000–353,000 vs. 244,000–306,000 platelets/µL), and WBC count (8180–8890 vs. 7100–7410 cells/µL). Parasite density in these two groups peaked between four to five years, then decreased steadily thereafter; conversely, haemoglobin showed a corresponding increase with age. Children with clinical malaria were markedly different: all had an average age below 5 years, had dramatically elevated parasite density (13,905–869,000 parasites/µL) and dramatically decreased platelet count (< 159,000 platelets/µL) and haemoglobin (< 7 g/dL).

**Conclusions:**

eBL and asymptomatic parasitaemia/antigenaemia, but not clinical malaria, were the most similar conditions with respect to mean age and malaria-related laboratory results. These results suggest that children with asymptomatic parasitaemia/antigenaemia may be the population at risk of eBL.

## Background

Endemic Burkitt Lymphoma (eBL) is an aggressive B-cell non-Hodgkin lymphoma that is associated with endemic *Plasmodium falciparum* malaria [1]. Thus, the incidence of eBL correlates with the endemicity of *P. falciparum* malaria [1–3] and eBL incidence is highest in malaria endemic countries in sub-Saharan Africa [4], where eBL cases account for 50–75% of childhood cancers in some countries [5]. The role of malaria is supported by significant associations of eBL risk with high antibody titers of markers of long-term exposure to *P. falciparum* infection [6–8] and inverse associations with antibodies that are associated with protection from severe *P. falciparum* infection [6, 9]. In addition, there is support from indirect evidence based on inverse associations with on carriage of genetic variants that are associated with resistance to severe malaria morbidity [10–12], especially the sickle cell trait [13]. However, these results, although important because they are not affected by reverse causality, have not been consistent because they were non-significant in some studies [14, 15] and null in at least one study [16]. The conflicting results may be due to small and under-powered studies or reliance on hospital-based studies or selection bias of controls.

Whether the relationship between malaria and eBL is related to malaria morbidity and circulating malaria parasite burden and inflammation [17], for which morbidity is a surrogate of, is unknown. The severity of clinical malaria (severe malaria anaemia, hyperparasitaemia, cerebral malaria, malaria prostration, moderate malaria, and mild malaria) is directly related to parasite burden and associated host response [18], but the correlation between clinical malaria, which is a surrogate for uncontrolled parasite burden [17], has not been investigated.

This paper reports an investigation to assess the patterns of age and selected malaria-related laboratory measures (parasite density, haemoglobin, platelet count, and white cell count (WBC) count) in children with eBL, asymptomatic parasitaemia/antigenaemia, and clinical malaria in Uganda, Tanzania, and Kenya using primary data from a case–control study or secondary data extracted from papers published in malaria endemic areas.

## Methods

### Sources of subjects evaluated for malaria-related laboratory measures

The analysis utilized primary data compiled from children enrolled in the Epidemiology of Burkitt Lymphoma in East African Children and Minors (EMBLEM) Study conducted in Uganda, Tanzania, and Kenya during 2010–2016 [19]. The EMBLEM study enrolled children aged 0–15 years old with eBL (histologically or cytologically confirmed in 61.4% of cases) at six local district or regional hospitals in Uganda, Tanzania, and Kenya [19]. EMBLEM also enrolled healthy children from 300 random villages in the same regions as the cases [19]. To ensure the comparability of malaria exposure before enrolment for the controls and eBL cases, eligibility was restricted to usual residents (≥ 4 months prior to enrollment) of the study area. Malaria was assessed on venous blood specimens using light microscopy to detect asexual malaria parasite forms and commercial malaria rapid diagnostic tests (RDTs) to detect histidine rich protein-2 (HRP-2) and pan-lactate dehydrogenase (pan-LDH) malaria antigens [20, 21], which remain detectable for 35–42 days after treatment of symptomatic malaria [22]. Asexual parasites were quantified against 200 white blood cells and normalized to parasites per µL of blood.

**Fig. 1:**
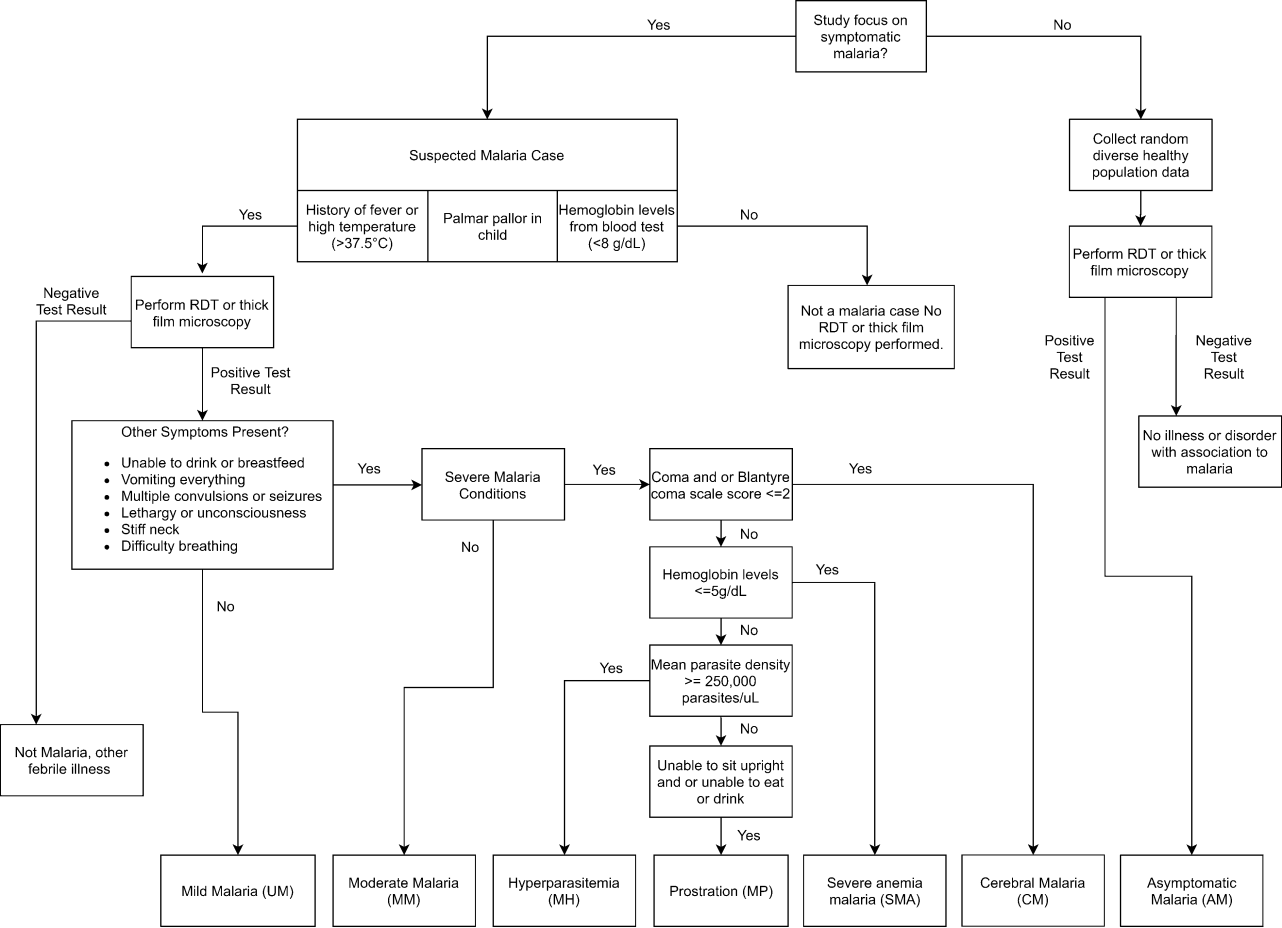
Flowchart of the stepwise evaluation of different malaria conditions

Secondary data on children with eBL or clinical malaria were compiled from published papers by searching PubMed and Google Scholar to identify representative papers about eBL and malaria conditions. Malaria conditions in these papers were classified according to the World Health Organization algorithm into asymptomatic parasitaemia/antigenaemia or clinical malaria with six conditions (severe malaria anaemia, hyperparasitaemia, cerebral malaria, malaria prostration, moderate malaria, and mild malaria; Fig. 1) [18]. The articles were identified using the following search terms applied in different combinations: Burkitt lymphoma, severe malaria, uncomplicated malaria, symptoms, malaria progression, coma, hyperparasitaemia, prostration, haemoglobin, platelets, parasite count, sub-Saharan Africa, anaemia, malaria, and country names: Uganda, Tanzania, and Kenya. The identified articles were retrieved and screened by LSR, who read the titles, abstracts, and when this was not sufficient, read the full text to identify reports with quantitative data about eBL or malaria conditions. Case reports, case series, or viewpoints were excluded. The papers were prioritized according to sample size, detail and completeness of data, and whether reporting data from Uganda, Tanzania, and Kenya. This list was reviewed by LSR and SMM to select, by consensus, representative papers for data extraction.

The selected papers were used as a source of data about children with eBL, asymptomatic parasitaemia/antigenaemia, and clinical malaria. The data elements, including demographics (age, gender, and mortality), symptoms (seizures, headaches, weakness, diarrhoea, myalgia, and vomiting), signs (fever, cough, coma, hepatosplenomegaly, jaundice, pallor, and respiratory distress), and malaria-related laboratory measures (complete blood count, including haemoglobin, platelets count, WBC count; parasite density, and malaria parasitaemia based on microscopy of thick blood smears or antigenaemia based on RDT), were extracted into a spreadsheet. Malaria parasite density > 250,000 parasites/µL of blood was used to define hyperparasitaemia; haemoglobin < 5 g/dL of blood was used to define severe malaria anaemia [18]; thrombocytopenia was defined as platelet counts < 150,000/µL [3]; while WBC count > 10,000 cells/µL was used to define leukocytosis, a general marker of inflammation.

### Statistical analysis

Results expressed on a continuous scale were extracted as means and standard deviations or as medians and inter-quartile ranges (IQR). Medians were statistically converted into means using standard statistical methods [23] to enable all comparisons to be made with means as the measure of central tendency. The results were checked for outlier values, which were excluded as appropriate. Results expressed as categories were extracted as the percent of subjects with values falling within a certain range. Data were analysed using R 3.6.0 and R Studio 1.2.1335. The patterns of mean age and the means of malaria-related laboratory measures in children with eBL, asymptomatic parasitaemia/antigenaemia, or clinical malaria were assessed descriptively. Regression and spline models were run to explore trends of parasite density, haemoglobin, platelet count, and WBC count across single-year age groups in children with eBL and asymptomatic malaria parasitaemia/antigenaemia in the EMBLEM study. Two-sided *P* < 0.05 with Bonferroni adjustment for the number of comparisons performed were considered statistically significant.

## Results

### Summary characteristics of study population

**Fig. 2:**
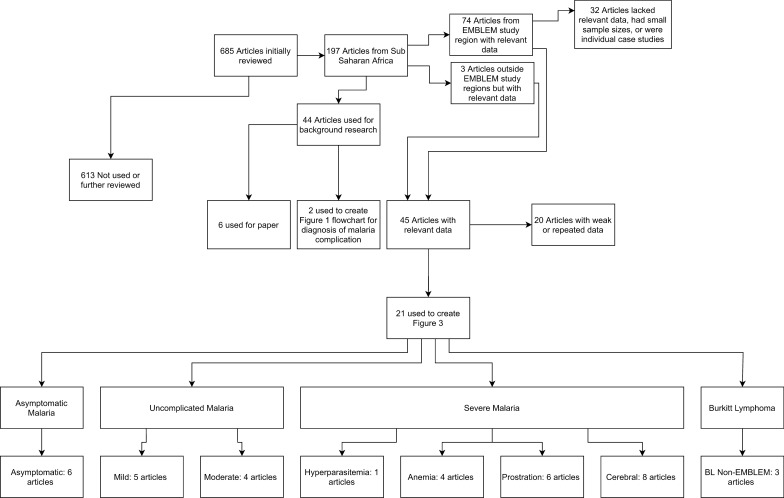
Flowchart showing the pipeline used to search and evaluate literature with relevant data

Results were available for 850 eBL cases and 2878 healthy controls with primary data in the EMBLEM study and 4019 eBL cases and 37,089 of 80,532 children with secondary data in 21 published articles selected from 685 articles had asymptomatic malaria infection or clinical malaria and their data were extracted (Fig. 2; Additional file 1: Table S1). Table 1 summarizes the patterns of demographics, parasite density, haemoglobin, platelet count, and WBC count for children with eBL, asymptomatic parasitaemia/antigenaemia, or six clinical malaria conditions. Children with eBL and asymptomatic parasitaemia/antigenaemia were similar in terms of their mean age (7.1–7.2 vs. 7.4–9.8 years), mean haemoglobin (10.1–10.4 vs. 11.7–12.3 g/dL), average malaria parasitaemia/antigenaemia prevalence (26.0–46.0% vs. 38.4–43.8%), parasite density (2800 vs. 1827–7780 parasites/µL), mean platelet count (347,000–353,000 vs. 244,000–306,000 platelets/µL), and WBC count (8180–8890 vs. 7100–7410 cells/µL). Children with eBL and asymptomatic malaria parasitaemia/antigenaemia were markedly different from those with all clinical malaria conditions, who were much younger, with their mean age below age 4 years, and had markedly abnormal malaria-related laboratory results (Table 1). Children with severe malaria anaemia and malaria hyperparasitaemia had a mean age less than 2 years; those with moderate malaria, malaria prostration, and cerebral malaria had a mean age between 2 to 3 years; and those with mild malaria had a mean age of 3.2 years. Furthermore, while 75% of children with severe anaemia or moderate malaria, 65% of those with malaria prostration, and 50% of those hyperparasitaemia, cerebral malaria, or mild malaria were in children aged 0 to 2 years, only 0.1% of children with eBL and 2.4% of children with asymptomatic parasitaemia/antigenaemia were in children in this age group. However, in contrast to malaria conditions in which the male-to-female ratio was similar for children with malaria (48% to 57% of children were male), those with eBL showed male predominance (61–63% were male).

**Table 1:** Patterns of symptoms, clinical signs and laboratory biomarkers found in malaria conditions in the literature and the EMBLEM Study

Type of malaria	Severe malaria		Uncomplicated malaria	Severe malaria		Uncomplicated malaria	Non-EMBLEM data		EMBLEM data	
Malaria conditions^a^	Anaemia	Hyperparasitaemia	Moderate malaria	Prostration	Cerebral	Mild malaria	Burkitt lymphoma	Asymptomatic controls	Burkitt lymphoma (n = 850)	Healthy controls (n = 2878)
Demographics	–	–	–	–	–	–	–	–	–	–
Age, years (mean + SD)	1.73 (1.73)	1.88 (1.94)	2.21 (1.25)	2.29(2.27)	2.64 (2.59)	3.22 (3.78)	7.1 (2.9)	9.8 (2.2)	7.16 (3.57)	7.39 (3.32)
0–2	76.8%	52.0%	73.5%	65.5%	55.4%	48.0%	2.4%	0.1%	10.1%	6.7%
3–5	22.5%	46.3%	26.4%	29.0%	34.8%	28.7%	30.6%	4.07%	34.8%	35.4%
6–8	0.7%	1.7%	0.1%	5.3%	9.1%	16.8%	38.6%	31.5%	24.9%	29.6%
9–11	0.00%	< 0.01%	0.01%	0.2%	0.7%	6.4%	18.0%	48.5%	16.7%	14.6%
12–15	0.00%	0.00%	< 0.01%	0.0%	0.0%	0.0%	10.4%	15.9%	13.4%	13.7%
Gender	–	–	–	–	–	–	–	–	–	–
Male	52.9%	51.4%	56.5%	52.5%	48.3%	51.7%	63.2%	47.8%	61.7%	53.4%
Female	47.1%	48.6%	43.5%	47.5%	51.7%	48.3%	36.8%	52.2%	38.3%	46.6%
Male:Female Ratio	1.12:1	1.06:1	1.30:1	1.11:1	0.93:1	1.07:1	1.71:1	1.15:1	1.61:1	1.15:1
Mortality										
% deaths from condition	2.7%	7.20%	5.00%	6.70%	26.70%	< 1%	15.69%	< 1%^a^	–	–
Malaria symptoms										
Multiple Seizures	4.7%	26.0%	1.99%	60.00%	78.00%	0.00%	–	0.00%	–	–
Headaches	–	–	88.00%	0.00%	12.00%	–	–	0.00%	30.52%	4.34%
Weakness/Lethargy	–	27.4%	14.00%	100.00%	12.40%	–	–	0.00%	66.18%	1.50%
Diarrhoea	40.6%	–	43.00%	–	6.10%	35.00%	–	0.00%	1.72%	0.53%
Myalgia	–	–	11.00%	0.00%	0.00%	–	–	0.00%	20.34%	0.04%
Nausea/vomiting	64.1%	–	58.00%	–	36.90%	39.00%	–	0.00%	17.28%	1.64%
Malaria signs										
Fever (> 37.5 °C)	–	–	–	–	–	–	–	–	–	–
Temperature (mean + SD)	37.3 (1.5)°C	37.9 (1.6)°C	37.5 (2.16)°C	38.0 (1.4)°C	37.94 (1.41) °C	37.7 (2.75)°C	–	36.3 (–)°C	–	–
% fever, last 6 months	56.5%	60.6%	51.00%	64.00%	62.30%	56.52%	–	0.00%	62.01%	70.93%
Dry cough	33.0%	–	36.00%	–	39.55%	26.00%	–	0.00%	26.23%	12.88%
Coma	0% (5)	0% (5)	0% (≥ 4)	0% (≤ 3)	98% (≤ 2)	0% (5)	–	0% (5)	0% (5)	0% (5)
Hepatosplenomegaly	68.0%	63.0%	13.00%	22.90%	55.00%	< 1%	–	0.00%	59.80%	0.24%
Jaundice	83.3%	24.0%	1.49%	28.60%	86.70%	4.10%	–	0.00%	11.64%	0.63%
Pallor	37.5%	24.0%	42.00%	–	34.55%	4.00%	–	0.00%	–	–
Respiratory distress	61.0%	71.0%	7.67%	100.00%	72.00%	7.00%	–	0.00%	33.09%	0.67%
Haemoglobin (HB), g/dL										
Hb (mean + SD)	3.8 (1.3)	4.9 (3.1)	7.8(2.38)	10.0 (3.3)	6.76 (3.24)	11.5 (0.8)	10.35 (3.24)	11.7(1)	10.05 (2.30)	12.26 (1.56)
Anaemia										
Any (< 11 g/dL)	100.0%	97.4%	91.01%	61.80%	90.40%	26.19%	57.80%	23.88%	63.42%	17.84%
Mild (8.0–10.9 g/dL)	0.06%	13.7%	37.83%	34.67%	25.62%	26.19%	34.47%	23.87%	43.97%	16.47%
Moderate (5.1–7.9 g/dL)	17.6%	32.6%	41.23%	20.64%	35.40%	0.00%	18.38%	0.01%	18.49%	1.29%
Severe (< 5 g/dL)	82.3%	51.1%	11.95%	6.49%	29.38%	0.00%	4.95%	0.00%	0.96%	0.08%
Platelet count										
Platelets (mean + SD) × 10^3^)	126.0 (109.0)	88.1 (85.3)	159.0(143.8)	100.7 (127.9)	82.5 (94.1)	247(168)	347.7 (220.6)	244.2 (135.3)	353.4 (184.3)	306.5 (117.7)
Normal (150,000–300,000 Platelets/µL)	41.3%	23.4%	52.40%	35.00%	23.65%	71.82%	81.50%	74.94%	88.24%	94.83%
Low (< 150,000 Platelets/µL)	58.7%	76.6%	47.60%	65.00%	76.35%	28.18%	18.50%	25.06%	11.76%	5.17%
White blood cell (WBC)										
WBC (Mean + SD) (× 10^3^)	13.3 (11.1)	12.3 (10.1)	10.3 (5.19)	8.3 (5.5)	10.31 (7.79)	13.5 (8.1)	8.18 (4.12)	7.1 (4.33)	8.89 (6.96)	7.41 (3.29)
High (> 10,000 cells/µL)	66.0%	62.0%	52.3%	38.1%	46.0%	66.7%	33.4%	25.1%	26.6%	12.9%
Normal (< 10,000 cells/µL)	34.0%	38.0%	47.7%	61.9%	54.0%	33.3%	66.6%	74.9%	73.4%	87.0%
Malaria parasite tests										
Malaria RDT, %	–	–	–	–	–		46%	38.43%	25.98%	43.81%
Parasite density (parasites/µL)										
Mean density + SD) × 10^3^)	49.4 (99.4)	869.3 (1166.2)	30.34(47.06)	44.6 (74.4)	38.9 (103.8)	13.905 (38.49)		1.827 (4.610)	2.81 (13.47)	7.78 (34.61)
Density < 250,000/uL)	97.8%	0.0%	100.0%	99.7%	97.9%	100.0%		100.0%	100.0%	99.6%
Density > 250,000/uL)	2.18%	100.0%	0.0%	0.29%	2.10%	0.00%		0.0%	0.0%	0.39%

^a^Malaria conditions are sorted in ascending order according to the age of diagnosis; Numbers are not included for non-EMBLEM subjects because these data were compiled from different papers (details in Additional file 1: Table S1)

### Malaria-related laboratory results differ by age group and condition

**Fig. 3:**
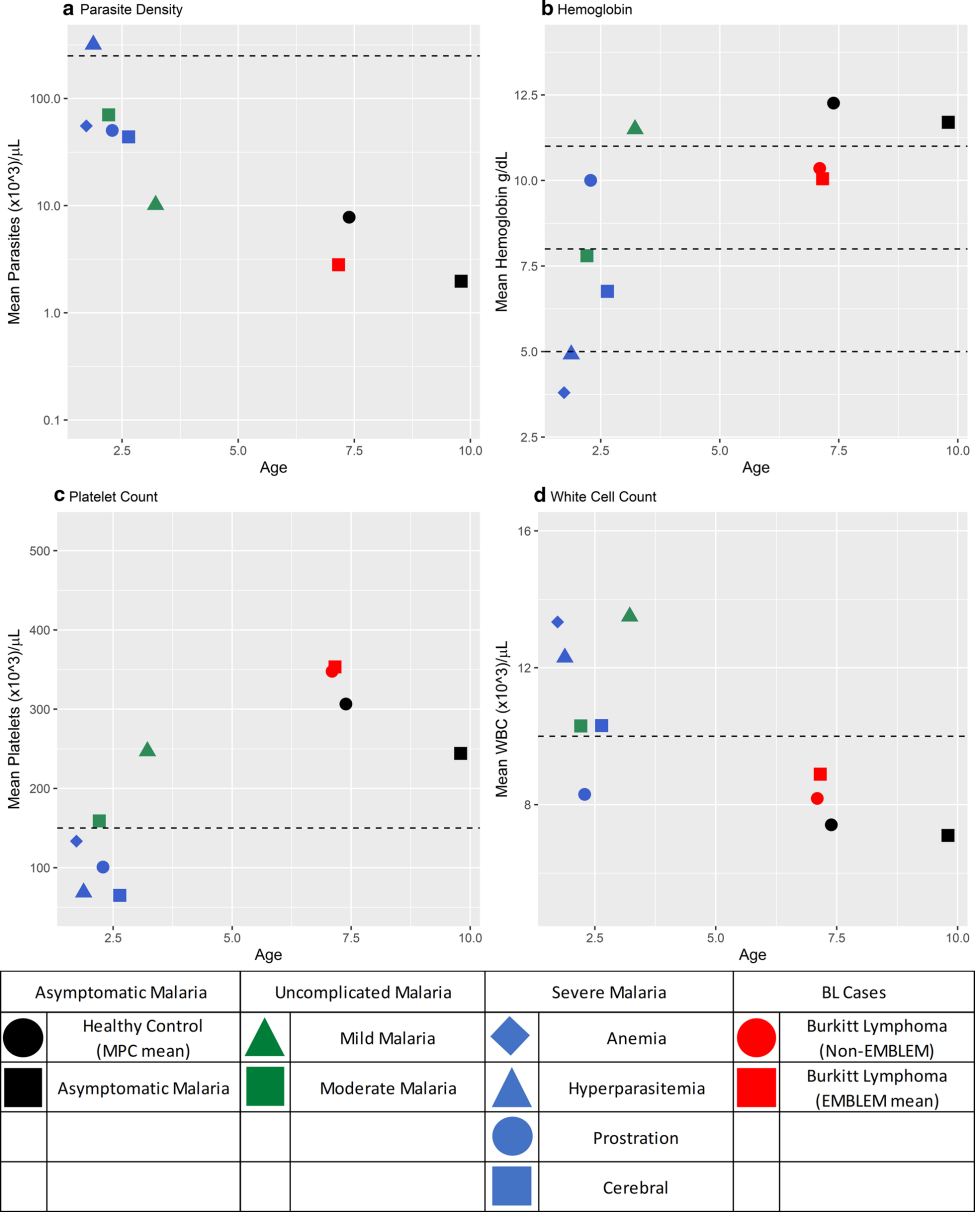
Multi-panel graph showing the mean values of malaria-related laboratory measures (parasite density, haemoglobin, platelet count, and white cell count) plotted according to the mean age of the malaria condition using data obtained from the literature and the EMBLEM Study. Dotted lines mark cut-off value for hyper-parasitaemia (**a**), for mild (11.0 g/dL), moderate (7.5 g/dL), and severe anaemia (< 5.0 g/dL) (**b**), for the lower range of platelet counts (**c**), and for elevated white blood cell count (**d**)

Malaria-related laboratory results showed distinct clusters in children below or above 5 years and by malaria condition (Fig. 3a–d). The children with eBL and asymptomatic parasitaemia/antigenaemia clustered above 5 years and has comparable average parasite density < 10,000 parasites/µL (Fig. 3a), haemoglobin ≥ 10.0 g/dL (Fig. 3b), platelet counts between 240,000 and  350,000 platelets/µL (Fig. 3c) and WBC count < 10,000 WBCs/µL (Fig. 3d). By contrast, the other malaria conditions clustered below 5 years and formed several sub-clusters. Considering parasite density, children with severe malaria anaemia, cerebral malaria, malaria prostration, moderate malaria, and mild malaria had more comparable parasite density between 10,000 to 100,000 parasites/µL, while those with hyperparasitaemia clustered apart with > 250,000 parasites/µL (Fig. 3a). Considering haemoglobin, three sub-clusters were apparent (Fig. 3b): children with mild malaria with haemoglobin of > 10 g/dL; those with cerebral and moderate malaria with haemoglobin of > 5 g/dL but < 10 g/dL; and those with severe anaemia and hyperparasitaemia with haemoglobin < 5 g/dL. When platelet counts were considered, three sub-clusters were observed (Fig. 3c). The children with severe anaemia, hyperparasitaemia, prostration, or cerebral malaria clustered below 150,000 platelets/µL; those with moderate malaria clustered around ~ 150,000/µL and those with mild malaria clustered ~ 250,000 platelets/µL. Considering leukocytosis (> 10,000 white blood cell count/µL), two sub-clusters of severe anaemia, hyperparasitaemia, mild malaria with WBC counts > 12,000 cells/µL; those with cerebral malaria and moderate malaria with WBC counts with ~ 10,000 cells/µL, and those with prostration with WBC counts < 10,000 cells/µL.

### Trends in malaria-related laboratory results across single year ages groups

**Fig. 4:**
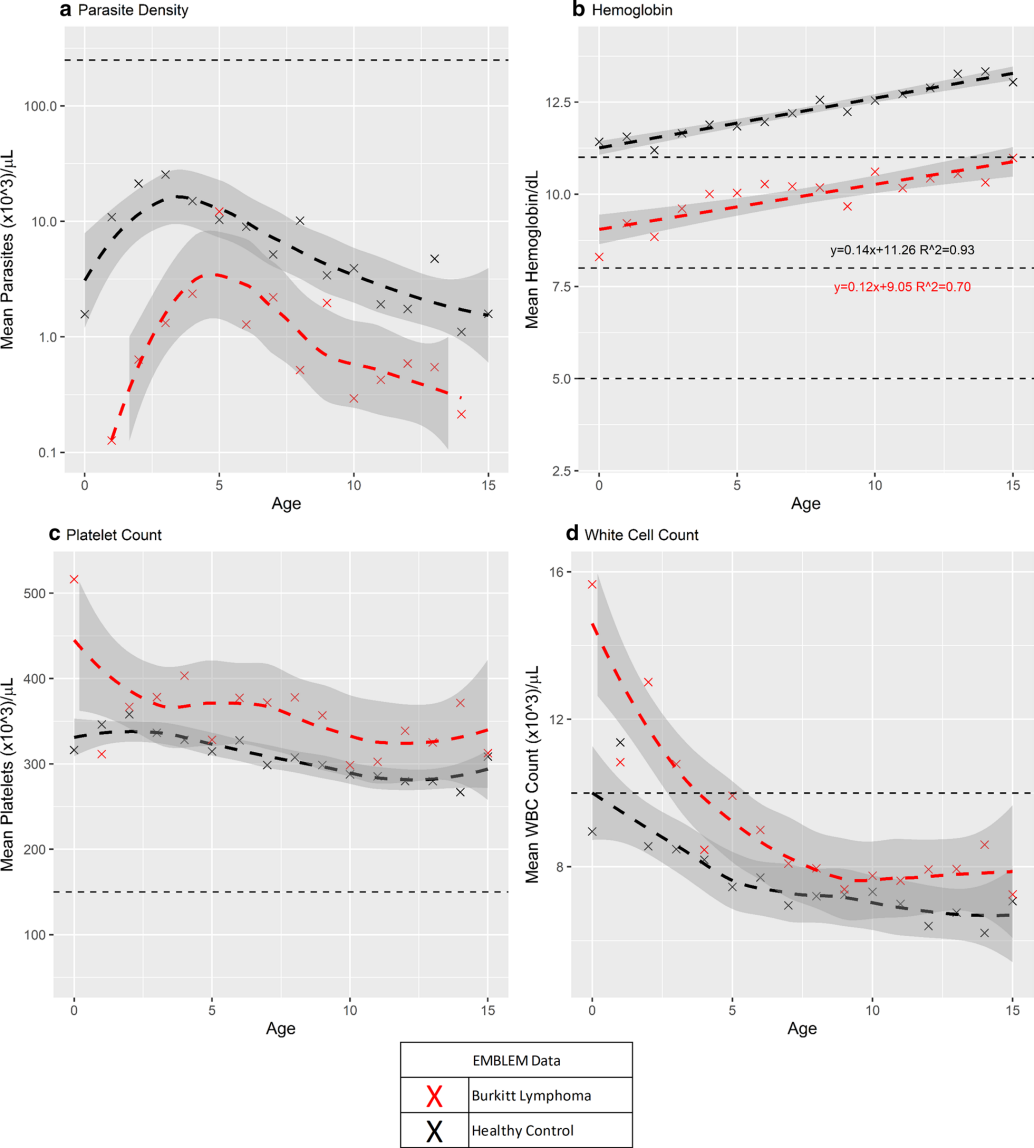
Multi-panel graph showing the modelled and raw age-specific mean values of malaria-related laboratory measures (parasite density, haemoglobin, platelet count, and white cell count) in eBL cases and children without eBL in the EMBLEM Study

Trends in parasite density, haemoglobin, platelet count, and WBC count across single year age groups in children with eBL or asymptomatic parasitaemia/antigenaemia in the EMBLEM study are shown in Fig. 4. Parasite density increased rapidly to peak around age 4 to 5 years in children with eBL and asymptomatic parasitaemia/antigenaemia, then decreased steadily thereafter in both groups (Fig. 4a). However, parasite density was significantly lower in children with eBL than those with asymptomatic parasitaemia/antigenaemia across all ages, with the difference being greatest in children below 5 years. The average haemoglobin was lower in children with eBL than those with asymptomatic parasitaemia/antigenaemia, but did not fall below 9 g/dL in the eBL cases and 11 g/dL in the children with asymptomatic parasitaemia/antigenaemia even in the youngest age group and it increased steadily with age (Fig. 4b). When platelet counts were considered, the count was greater than 300,000 platelets/µL in children with eBL and those with asymptomatic parasitaemia/antigenaemia, but the values being higher in the former than the later group (Fig. 4c). The WBC counts were generally similar in both groups, except in children below age 5 years in whom counts were slightly higher in the children with eBL (Fig. 4d).

### Overlap patterns of children with eBL, asymptomatic, and clinical malaria conditions

**Fig. 5:**
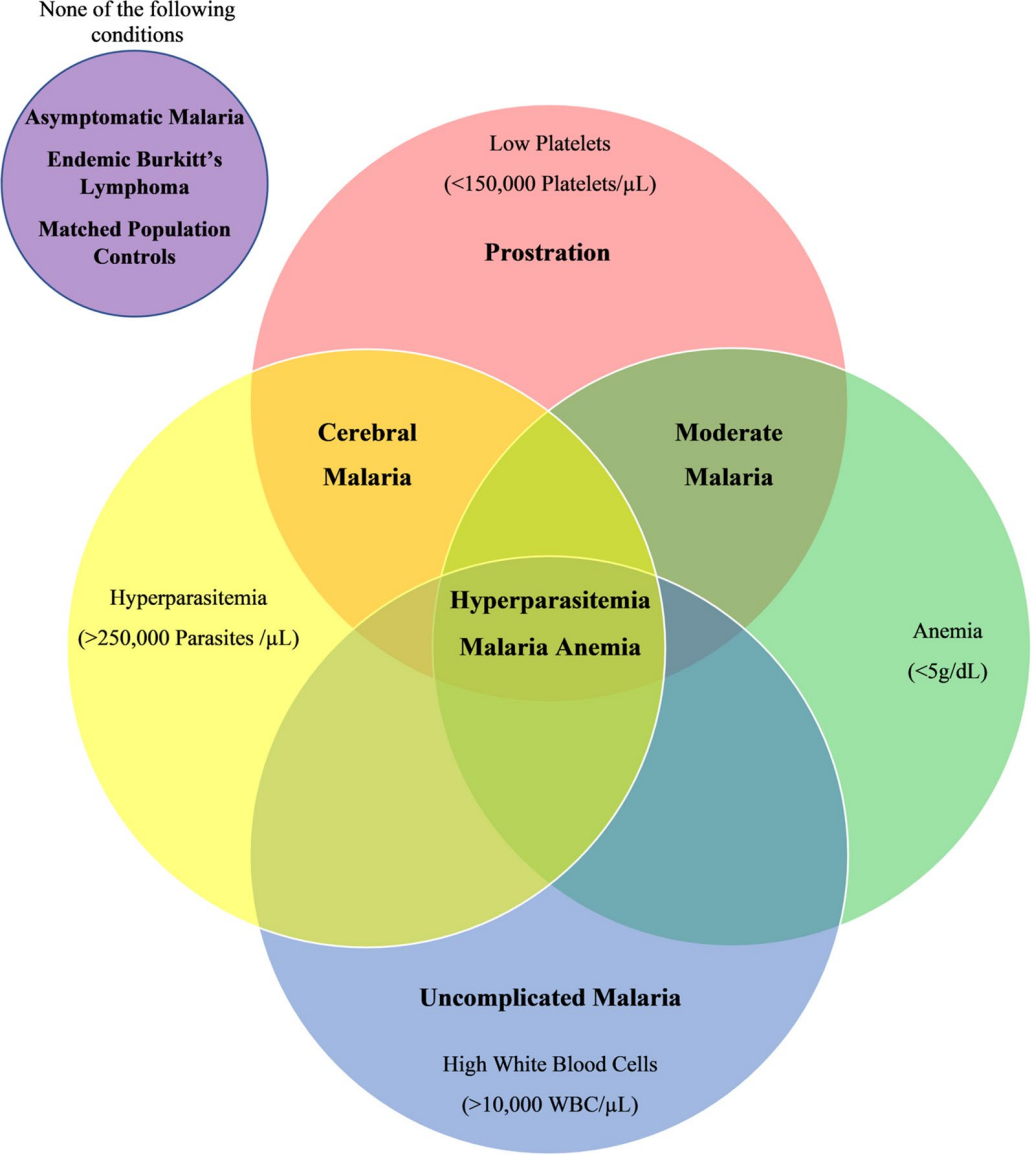
Discrete and overlapping malaria conditions based on mean age and biomarker levels (see text)

Figure 5 summarizes the two overlap patterns showing that children with eBL and asymptomatic parasitaemia/antigenaemia cluster together in children > 5 years and that both groups are characterized by normal or near normal laboratory values (low parasite density, mild anaemia, and normal platelet and WBC counts). Conversely, children with clinical malaria conditions cluster together below 5 years in three sub-clusters characterized by: (a) those with severe malaria anaemia and hyperparasitaemia with markedly abnormal values of parasite density, haemoglobin, platelet count, and WBC count; (b) those with cerebral malaria and moderate malaria characterized by abnormal values of platelet count and either parasite density or haemoglobin; (c) those with malaria prostration and mild malaria characterized by abnormal values of platelet count or WBC count.

## Discussion

The current study was done to evaluate whether eBL risk might be connected with high parasite density using acute malaria conditions as surrogates of moderate or high parasite density. The results show clustering of eBL and asymptomatic parasitaemia/antigenaemia in children aged > 5 years who have normal or near normal values of parasite density, haemoglobin, platelet, and WBC counts. These results suggest that eBL could be a complication of asymptomatic parasitaemia/antigenaemia and cast doubt on the idea that it is a complication of high parasite density or the associated high inflammation, as was originally hypothesized. High parasite density was a feature of three sub-clusters of clinical malaria, all found in children below 5 years. These early age-onset malaria syndromes were characterized by markedly abnormal values of parasite density, haemoglobin, platelet counts and WBC counts. These syndromes included children with severe malaria anaemia and hyperparasitaemia, those with cerebral malaria and moderate malaria, and those with malaria prostration and mild malaria and markedly abnormal values of some or all of parasite density, haemoglobin, platelet counts and WBC counts. The findings in this study are likely valid because ~ 9.5% of eBL cases and 6.7% of the controls in the EMBLEM study were 0–3 years, but even in this age range the children with eBL and asymptomatic parasitaemia/antigenaemia still showed malaria-related laboratory measures that were different from those in children with acute malaria conditions. Moreover, children with eBL had significantly lower parasite density than those with asymptomatic parasitaemia/antigenaemia suggesting that the similarity of eBL with asymptomatic parasitaemia/antigenaemia observed in overall results is established at a very early age. These results are consistent with a recent review by Quintana et al. [24] where they propose an explanation of how malaria exposure may precipitate the malignant transformation of a B-cell clone that may progress to eBL. Together, the accumulating evidence suggests that eBL develops in children who control malaria parasitaemia well, perhaps due to acquired age-related immunity to malaria [25], and that the capacity to control malaria parasitaemia precedes and continues after eBL onset.

Antigenic stimulation from malaria is widely accepted as an insult that leads to B cell proliferation in the germinal center and increases the chance of translocation of *c*-*MYC* into the vicinity of immunoglobulin enhancer elements and progression to eBL [26]. However, it has remained unclear whether uncontrolled malaria parasite proliferation, i.e., high parasite density, plays a key part in antigenic stimulation from malaria that triggers eBL development. The analysis presented suggests otherwise because asymptomatic parasitaemia/antigenaemia is associated with well-controlled malaria infection. If so, then children with eBL have a well-controlled malaria infection at the time of their diagnosis. The results from EMBLEM are notable because previous analyses suggest that eBL cases were more likely to be exposed to heavy malaria, based on being more likely to live in a village near surface water, to report inpatient or outpatient malaria history > 13 months before enrollment, than geographically matched controls [12], thereby underscoring the likelihood that immunity to malaria is likely to develop prior to eBL onset. The current analysis suggests that immunity to malaria appears to be established from a young age because children below 5 years with eBL had lower haemoglobin than children with asymptomatic malaria, consistent with heavy exposure to malaria, but significantly lower parasite density, consistent with a capacity to control malaria parasitaemia in young children. In view of the fact that malaria is the pre-eminent cause of early mortality in areas where eBL is common [17], the overlap patterns observed support a speculation that eBL occurs in children who are adapted to heavy exposure to malaria and it may be a trade-off exchange for a high risk for death from acute malaria. Because children with eBL are less likely to carry the classical polymorphisms that protect from severe malaria [10–12], such as the sickle cell trait [12], the trade-off for the much rarer, albeit deadly at the individual level, like involves mechanisms of resistance to early malaria mortality that are currently unknown but worth investigating [12].

Asymptomatic malaria is typically associated with low-, rather than high-parasite density malaria parasitaemia. Thus, these results also suggest that eBL risk may be associated with low density malaria infection. Furthermore, because an asymptomatic infection, particularly in children above 5 years, is also likely to be a clonally diverse infection [27], these results raise the hypothesis that a clonally diverse malaria antigenic stimulation is related to eBL development. This hypothesis is consistent with previous findings that concurrent infection with multiple malaria genotypes was correlated with eBL [28] and that eBL cases were more likely to have a higher *P. falciparum* genetic diversity score than controls [29]. This hypothesis is also consistent with findings in the EMBLEM study that children with eBL were less likely to report clinical malaria up to 12 months before eBL diagnosis and less likely to have detectable malaria parasite/antigens at enrolment [19] because children aged > 5 years with clonally diverse malaria infection had a decreased risk for clinical malaria during a follow up of 90 or more days [30]. The hypotheses that parasite density and genetic diversity are related to eBL could be tested in the era of next generation sequencing or proteomic technologies [28, 29].

This study has some limitations, notably, reliance on cross-sectional data. Thus, the correlations should not be interpreted as evidence for causality. Second, the reliance on published literature to increase complement assessment in EMBLEM is a strength, but the literature may be biased or incomplete. For example, studies of asymptomatic parasitaemia/antigenaemia may under-sample children below 5 years, or studies of severe malaria may oversample children below 5 years, which would lead to erroneous mean age patterns. However, the results from the EMBLEM study, which enrolled representative, population-based eBL cases and controls and uniformly tested subjects for malaria-related laboratory measures, support the overall patterns. This study lacks direct measures of immunity to malaria, including anti-malaria antibodies, which limits the ability to compare the immune status of eBL cases with that of children with asymptomatic parasitaemia/antigenaemia or clinical malaria. The strengths of this study include the large sample size and detailed data from the EMBLEM study and published data about children with malaria from malaria endemic areas.

## Conclusions

The findings suggest that children with eBL and asymptomatic parasitaemia/antigenaemia were most similar with respect to age and malaria-related laboratory results and that both groups were markedly different from children with clinical malaria. These results raise a testable hypothesis that asymptomatic parasitaemia/antigenaemia, and by extension its associated low malaria parasite density, may be the antigenic stimulation from malaria related to eBL onset.

## Supplementary information

**Additional file 1: Table S1.** A list of articles with used as sources for relevant data about malaria conditions in East Africa.

## Data Availability

The data and the code used for these analyses are available on reasonable requests from the corresponding authors.

## Abbreviations

OR: Odds ratio
CI: Confidence interval
eBL: Endemic Burkitt lymphoma
WBC: White blood cell
EMBLEM study: Epidemiology of Burkitt Lymphoma in East African Children and Minors Study
HRP-2: Histidine rich protein-2
pan-LDH: Pan-lactate dehydrogenase
